# Quarterly Report of Proceedings

**Published:** 1857-07

**Authors:** 


### QUARTERLY REPORT OF THE PROCEEDINGS.

*Monday, April 6th,* 1857; Dr. Babington in the chair. The Annual Report of the Council was read by Dr. McWilliam, and the office bearers for 1857-8 were elected.

The following papers were read:—

1. "On an Outbreak of Fever on Board the Ottoman Ship of the Line *Tesherefieh*;" and

2. "On Scurvy among the Crew of the Ottoman Brig *Fezri Sefet*." By J. N. Radcliffe, Esq., late of the Ottoman Medical Staff.

A discussion followed, in which Dr. Milroy, Dr. McWilliam, Dr. Greenhow, Mr. Kiallmark, Mr. Spencer Wells, and Dr. Babington took part.

---

*Monday, May 3rd.* The following papers were read by Dr. McWilliam.

1. "On the Cholera Epidemy, at Fogo, in the Cape de Verd Islands in 1855." By Jose Fernandez da Silva Leaō, Surgeon of the 1st class in the Portuguese Navy, and Colonial Surgeon of the Cape de Verd Archipelago.

2. "Extract from the Report of Dr. Agosthino Jose Ramos de Carvalho, Chief of the Medical Staff, and President of the Board of Health of the Cape de Verd Islands."

3. "Observations on the Epidemic Cholera, as it appeared at Rio de Janeiro, during 1855-6." By Geo. Lee, M.D., of Rio de Janeiro.

4. "On Cholera at Para in the Brazils, in 1855-6." By Samuel Vines, Esq., H.B.M. Consul at Para.

These papers were discussed by Dr. Waller Lewis, Dr. Milroy, Dr. Snow, Dr. Greenhow, Dr. Camps, Dr. Tripe, and Dr. Babington.

*Monday, June 1st.* "On the Mortality from Eruptive Fevers at different parts of the year." By J. W. Tripe, M.D. The paper was discussed by Dr. Babington, Dr. Milroy, Dr. Patrick Fraser, Mr. Marson, Dr. Tripe, and Dr. McWilliam.

---

### PRESENTATIONS.

The following donations to the Library of the Society have been made during the quarter:—

1. "Reports on the Sanitary Condition of the Whitechapel District, for the quarters ending December 27th, 1856, and March 30th, 1857. By John Liddle, Esq., Medical Officer of Health for the Whitechapel District." Presented by the Author.

2. "Gazette Médicale d'Orient, publiée par la Societé Imperiale de Médecine de Constantinople." Presented by the Society, through Dr. Babington.

3. "Des Hemorrhagies Pulmonaires, au point de vue de la Physiologie Pathologique, de l'Etiologie, et de la Semeiologie. Par M. J. D. Tholozan, Médecin-Major, Professeur Agregé à l'Ecole Imperiale de Médecine et de Pharmacie Militaires."

4. "Recherches sur les Maladies de l'Armée d'Orient, pendant l'Hiver de 1854 à 1855: lues à l'Académie de Médecine, dans sa séance du 30 Septembre, 1856. Par M. le Docteur Tholozan." Both these works were presented by the Author, through Dr. Waller Lewis.

---

### NEW MEMBERS.

*Corresponding.*—Wm. R. E. Smart, M.D., Staff Surgeon, R.N. Hospital, Hong Kong.

*Resident.*—Frederick John Burge, Esq., M.R.C.S., Hammersmith.

